# Application of Synthetic Microbial Communities of *Kalidium schrenkianum* in Enhancing Wheat Salt Stress Tolerance

**DOI:** 10.3390/ijms26020860

**Published:** 2025-01-20

**Authors:** Jing Zhu, Qiong Jia, Qi-Yong Tang, Ghenijan Osman, Mei-Ying Gu, Ning Wang, Zhi-Dong Zhang

**Affiliations:** Xinjiang Key Laboratory of Special Environmental Microbiology, Institute of Microbiology, Xinjiang Academy of Agricultural Sciences, Urumqi 830091, China; zhujing2020@hotmail.com (J.Z.); jiaqiong7270@163.com (Q.J.); tqy25@163.com (Q.-Y.T.); ghenijan85@foxmail.com (G.O.); gmyxj2008@163.com (M.-Y.G.); wangning950203@163.com (N.W.)

**Keywords:** *Kalidium schrenkianum*, synthetic microbial communities, wheat, salt stress tolerance

## Abstract

Soil salinization poses a significant challenge to global agriculture, particularly in arid and semi-arid regions like Xinjiang. *Kalidium schrenkianum*, a halophytic plant adapted to saline-alkaline conditions, harbors endophytic microorganisms with potential plant growth-promoting properties. In this study, 177 endophytic bacterial strains were isolated from *K. schrenkianum*, and 11 key strains were identified through functional screening based on salt tolerance, nutrient solubilization, and growth-promoting traits. Synthetic microbial communities (SMCs) were then constructed using these strains and optimized to enhance wheat growth under salt stress. The SMCs significantly improved seed germination, root length, and seedling vigor in both spring and winter wheat in hydroponic and pot experiments. Furthermore, the SMCs enhanced the activities of antioxidant enzymes, including superoxide dismutase (SOD), peroxidase (POD), catalase (CAT), and levels of malondialdehyde (MDA) and proline (PRO). They also reduced oxidative stress and improved chlorophyll content in wheat seedlings. These results demonstrate the potential of microbial consortia derived from extreme environments as eco-friendly biofertilizers for improving crop performance in saline soils, offering a sustainable alternative to chemical fertilizers and contributing to agricultural resilience and productivity.

## 1. Introduction

Soil salinization is a major ecological and environmental challenge for dryland agriculture worldwide, severely constraining agricultural development and sustainable growth [1,2]. Salinized lands are widely distributed across the globe, particularly in arid and semi-arid regions. According to data from the Food and Agriculture Organization of the United Nations (FAO), approximately 1 billion hectares of land are affected by salinization, accounting for about 7% of global arable land [3]. Xinjiang is known as a “museum of saline-alkali soils”, with salinized land covering approximately 2.81 × 10^7^ hectares, making up one-third of its arable area [4]. This region also has a rich diversity of halophytes, with species from the *Chenopodiaceae* family being the most prevalent. *Kalidium schrenkianum*, a halophytic plant belonging to the *Chenopodiaceae* family, is well adapted to moderate salinity levels [5]. This plant can absorb large amounts of salt ions from the environment, helping to improve saline-alkaline soil properties by reducing salt content and increasing organic matter.

Endophytic microorganisms, through long-term symbiotic interactions with plants [6], not only promote plant growth but also enhance their resistance to environmental stresses such as salinity, drought, and pests [7,8,9,10]. This study found that under drought stress, inoculation with two native endophytic species significantly improved the growth and stress tolerance mechanisms of endostemon obtusifolius, including increased production of osmotic regulating substances (soluble sugars, proline), upregulation of antioxidant enzyme systems (superoxide dismutase), and increased content of antioxidant metabolites (total phenols, flavonoids) [11]. Studies have also shown that endophytic bacteria play an important role in alleviating drought and salt stress in wheat by stimulating the production of plant growth regulators (PGRs) to alleviate the effects of adversity and improve plant growth [12]. Endophytes and rhizobacteria promote plant growth and yield under stress conditions. Utilizing valuable endophytes is critical for the environment, food, and sustainable agriculture. Therefore, studying the community structure of endophytes and their symbiotic mechanisms with plants is of great significance for ameliorating saline soils, breeding salt-tolerant crops, and advancing the utilization of biological resources.

The use of beneficial microbes to enhance plant growth conditions and increase crop yields has a long history in practice agriculture [13,14]. However, the effectiveness of single-strain inoculants is often limited by various environmental factors, leading to inconsistent functional outcomes [15,16,17]. Synthetic microbial communities (SMCs) are artificially constructed microbial assemblages, formed by co-cultivating two or more species under controlled conditions [18,19,20]. Compared to natural microbial communities and single-strain inoculants, SMCs have significant advantages: They simplify the complexity of microbial communities, exhibit greater adaptability to environmental variations, and enable more complex functionalities. Additionally, SMCs facilitate the development and validation of more intricate system models through mathematical modeling [18,21,22,23]. This versatility makes the use of SMCs a promising approach for improving agricultural sustainability and productivity.

Wheat is one of the world’s most important staple crops and a primary grain in China [24]. However, wheat cultivation faces significant challenges, particularly from abiotic stresses. Among these stresses, as emphasized by Godoy and Hossain, salinity poses a serious threat to wheat production, especially in arid and semi-arid regions [25,26]. Although extensive research has been conducted on the molecular mechanisms of wheat stress resistance, translating these findings into practical applications remains a considerable challenge [27,28,29,30]. Thus, re-programming wheat genetics to improve its adaptability to abiotic stresses through microbial intervention has emerged as a promising approach. Recent studies have highlighted this potential. For instance, Rauf [31] reported that inoculating waterlogged wheat with endophytic fungus *Trichoderma* MAP1 could mitigate the adverse effects of waterlogging by re-programming genes associated with polyamine (PAs) biosynthesis and enhancing the effects of exogenous ethephon (ET). Similarly, Shekhawat [32] reported that the root endophyte *Enterobacter* SA187 could induce heat tolerance in wheat, both in laboratory and field settings.

In this study, the core representative endophytic bacterial strains were isolated and screened from *K. schrenkianum* in the saline-alkaline environment of Xinjiang. A synthetic functional microbial community capable of countering salt stress was then developed. By facilitating cross-species colonization in wheat, the influence of this microbial consortium on wheat salt tolerance was further investigated. The aim of this work is to provide a theoretical basis for enhancing wheat yield and ecological restoration in saline-alkaline regions, laying the groundwork for the development and application of related microbial resources.

## 2. Results

### 2.1. Isolation and Identification of Strains

**Figure 1:**
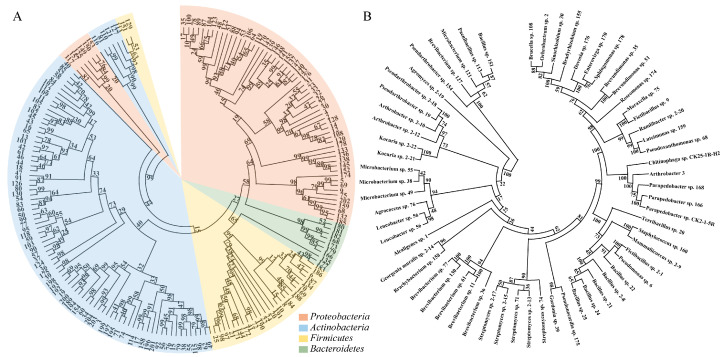
Phylogenetic tree of endophytic bacteria isolated from *Kalidium schrenkianum* based on 16S rRNA sequencing. The phylogenetic tree was constructed using the neighbor-joining method in MEGA 11 software. (**A**) Different colors represent taxonomic levels at the phylum; (**B**) genus-level classification is shown on the right side of the tree.

Using 16S rRNA gene sequencing, a phylogenetic tree illustrating the bacterial community structure was constructed, as shown in Figure 1. The phylogenetic analysis revealed the identification of 177 endophytic bacterial strains isolated from *K. schrenkianum*, belonging to 39 genera and 63 species (Table S1). These bacteria were classified into four major phyla: *Actinobacteria*, *Proteobacteria*, *Firmicutes*, and *Bacteroidetes*. Among them, *Actinobacteria* constituted 45.8% of the community, indicating its high abundance in the sample. This was followed by *Proteobacteria* (33.9%) and *Firmicutes* (16.9%), both of which also exhibited high diversity and abundance. In contrast, *Bacteroidetes* were relatively less abundant, accounting for only 3.4% (Figure 1A).

At the genus level, the phylogenetic tree demonstrated a highly diverse microbial community within the sample. The distinct clustering of different genera reflected their evolutionary divergence. Notably, genera such as *Alpha Proteobacterium*, *Bacillus*, *Brevibacterium*, *Microbacterium*, and *Streptomyces* exhibited high abundance (Figure 1B).

### 2.2. Screening of Growth-Promoting Strains

**Figure 2:**
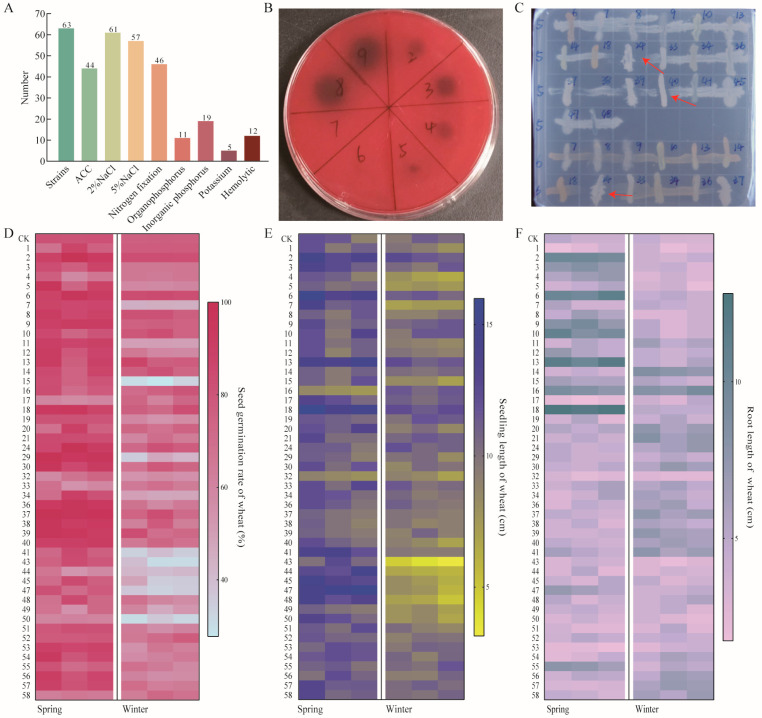
Screening of growth-promoting strains. (**A**) Functional characteristics of strains; (**B**) hemolytic activity; (**C**) strain antagonism, red arrows indicate strains that are antagonistic to each other; (**D**) effects of strains on seed germination rate of hydroponic wheat; (**E**) effects on seedling length; (**F**) effects on root length.

In this study, a total of 63 bacterial strains were isolated and subjected to a series of functional screenings, including salt tolerance (2% NaCl and 5% NaCl), nitrogen fixation, phosphate solubilization (both organic and inorganic), potassium solubilization, and acetyl-CoA carboxylase (ACC) deaminase activity. Among the isolates, 44 strains exhibited ACC deaminase activity, 61 strains were capable of growing under 2% NaCl condition, and 57 strains were able to grow under 5% NaCl condition. A total of 46 strains demonstrated nitrogen-fixing abilities. A total of 11 strains were able to solubilize organic phosphorus, while 19 strains were capable of solubilizing inorganic phosphorus. Additionally, 5 strains exhibited potassium solubilizing activity. Hemolytic activity was detected in 12 strains (Figure 2A,B). The antagonistic interactions among the 65 isolated strains were also evaluated. The results demonstrated that strain NO. 24 exhibited antagonistic effects against both strain NO. 5 and NO. 6, while strain NO. 5 also antagonized strain NO. 40 (Figure 2C).

Strains with strong salt-alkali tolerance, non-hemolytic properties, and no antagonistic interactions were selected for germination and hydroponic experiments using seeds of “Xin Chun 26” (spring wheat) and “Xin Dong 18” (winter wheat). Based on a comprehensive analysis of the effects of each strain on germination rate, root length, and seedling length in spring and winter wheat, 11 strains with significant growth-promoting effects were identified (Figure 2D–F, Table 1). Among them, two strains were classified within the genus *Bacillus*, while the remaining nine strains were derived from various other genera.

### 2.3. Construction of SMCs

**Figure 3:**
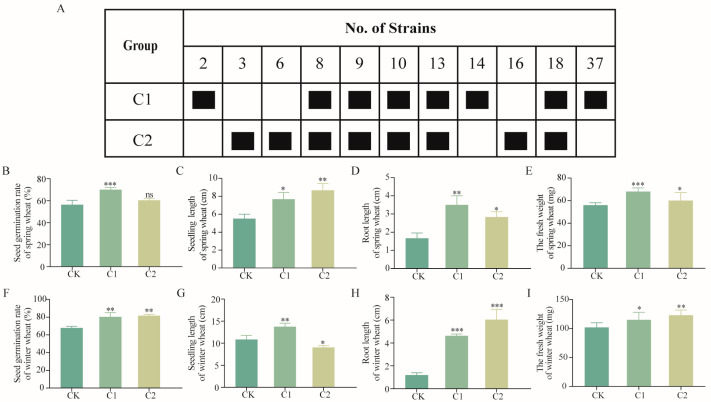
Construction and effects of SMCs on wheat growth. (**A**) Strain composition of C1 and C2; (**B**–**E**) effects of C1 and C2 on germination rate, seedling length, root length, and fresh weight of spring wheat; (**F**–**I**) effects of C1 and C2 on germination rate, seedling length, root length, and fresh weight of winter wheat. CK: wheat seeds soaked in sterile water; C1 and C2: wheat seeds soaked in synthetic microbial cultures with an OD_600_ of 0.2. Data represent mean ± standard error (*n* = 8) and were analyzed using one-way ANOVA, ns, *p* > 0.05, *, *p* < 0.05; **, *p* < 0.01; ***, *p* < 0.001.

11 strains were randomly combined to form various synthetic communities, leading to the identification of two effective combinations, C1 and C2 (Figure 3A). C1 increased the germination rate of spring wheat by 24%, seedling height by 39%, root length by 100%, and fresh weight by 21% (Figure 3B–E). At the same time, C1 demonstrated a strong growth-promoting effect on winter wheat. Compared with the control group, C1 increased the germination rate of winter wheat by 18%, seedling length by 27%, root length by 300%, and fresh weight by 13% (Figure 3F–I). In contrast, C2 showed no significant effect on the germination rate of spring wheat (*p* > 0.05), and had a significant promoting effect on seedling length, root length, and fresh weight (*p* < 0.01, *p* < 0.05, *p* < 0.05), but the promoting effect on root length and fresh weight were weaker than that of C1 (Figure 3B–E). For winter wheat, C2 increased the germination rate by 20%, root length by 500%, and fresh weight by 21% (Figure 3F–I).

### 2.4. Optimization of SMCs

**Figure 4:**
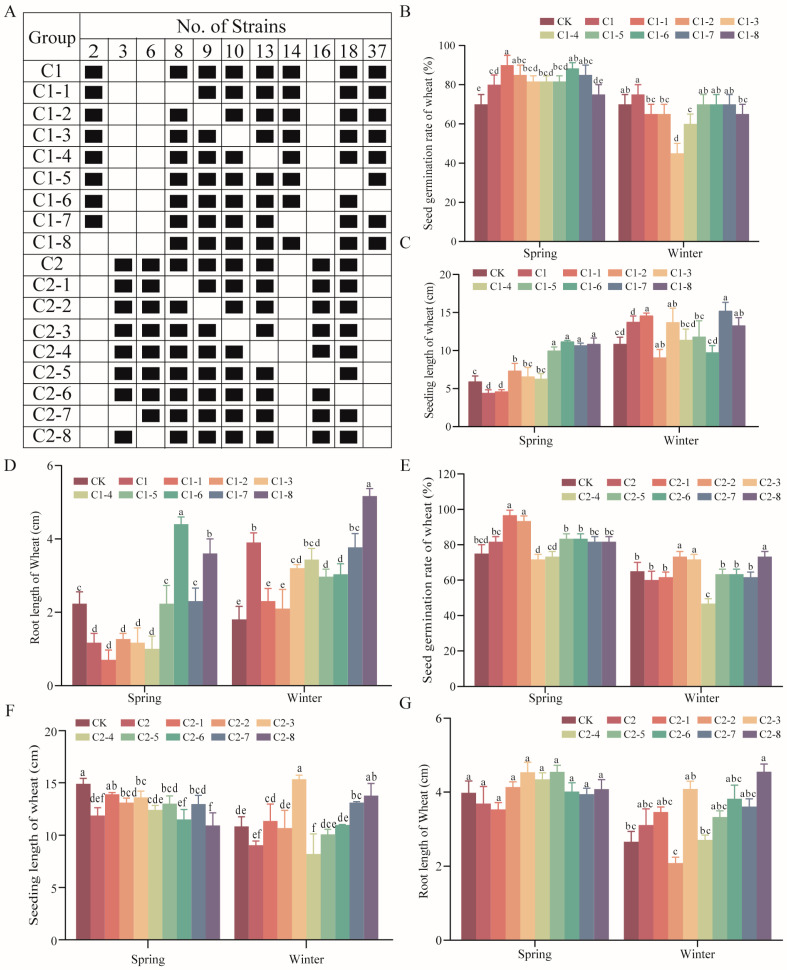
Optimization of SMCs. (**A**) Composition of C1, C2, and their derivative combinations; (**B**–**D**) effects of C1 and its derivatives on germination rate (**B**), seedling length (**C**), and root length (**D**) of spring and winter wheat; (**E**–**G**) effects of C2 and its derivatives on germination rate (**E**), seedling length (**F**), and root length (**G**) of spring and winter wheat. Data represent mean ± standard error (SE) and different letters indicate significant differences (*p* < 0.05, Tukey’s test, *n* = 15).

Under hydroponic conditions, the effects of 2 SMCs, C1 and C2, and their derivative combinations on the germination rate, seedling length, and root length of spring wheat and winter wheat were evaluated. In the spring wheat treatment experiments, C1 and its combinations (e.g., C1-6, C1-3) significantly enhanced seed germination rates (Figure 4B) and seedling lengths (Figure 4C), with the most pronounced effect observed in the C1-6 treatment. Notably, C1-6 significantly promoted both seedling length (Figure 4C) and root length (Figure 4D) of spring wheat, increasing these metrics by approximately 98% and 97%, respectively, compared to the control group. Among the C2 and its combinations, C2-1 markedly improved germination rates and seedling lengths in spring wheat (Figure 4E,F), showing significantly better performance than other combinations.

For winter wheat, the C1-8 treatment demonstrated superior performance in germination rate, seedling length, and root length. Meanwhile, C2-3 was the most effective treatment for winter wheat, showing significant enhancement in germination rate and root length (Figure 4G). Together, these results highlight the superior efficacy of C1-6 and C2-1 for spring wheat and C1-8 and C2-3 for winter wheat.

### 2.5. Effects of SMCs on Wheat Potted Plants

**Figure 5:**
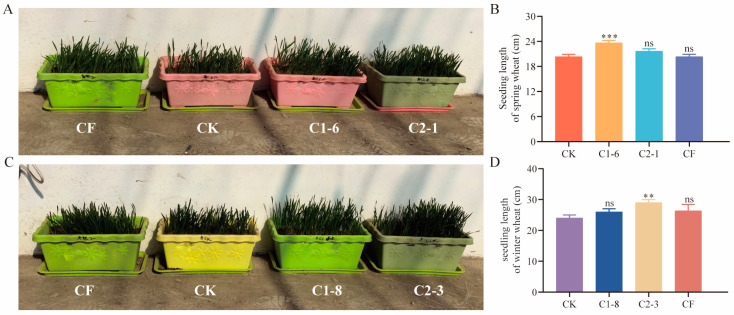
Effects of SMCs on potted wheat plants. (**A**) Morphological effects of C1-6 and C2-1 treatments on spring wheat; (**B**) seedling length of spring wheat under C1-6 and C2-1 treatments; (**C**) morphological effects of C1-8 and C2-3 treatments on winter wheat; (**D**) seedling length of winter wheat under C1-8 and C2-3 treatments. CK: seeds treated with water; CF: seeds treated with commercial fertilizer; C1-6, C2-1, C1-8, and C2-3: seeds treated with the respective bacterial solutions. Data represent mean ± standard error (*n* = 30) and were analyzed using one-way ANOVA, ns, *p* > 0.05; **, *p* < 0.01, ***, *p* < 0.001.

In the spring wheat pot experiment, treatments with commercially available fertilizer (CF), C1-6, and C2-1 significantly increased the germination rate of spring wheat seeds compared to the control group (Figure 5A). The seedling length measurements showed that C1-6 significantly promoted the growth of spring wheat seedlings (*p* < 0.001), while no significant growth promotion was observed for C2-1 and CF treatments (*p* > 0.05). This suggested that C1-6 had a superior growth-promoting effect on spring wheat (Figure 5B).

In the winter wheat pot experiment, treatments with CF, C1-8, and C2-3 also enhanced the germination rate of winter wheat seeds (Figure 5C). Further analysis of seedling length revealed that C2-3 significantly promoted the growth of winter wheat seedlings, increasing their length by 1.2 times compared to the control group (*p* < 0.01, Figure 5D). In contrast, no significant differences in seedling length were observed for CF or C1-8 treatments (*p* > 0.05). Therefore, C2-3 exhibited a more pronounced growth-promoting effect for winter wheat.

### 2.6. Effect of SMCs on Enzyme Activity in Potted Wheat

**Figure 6:**
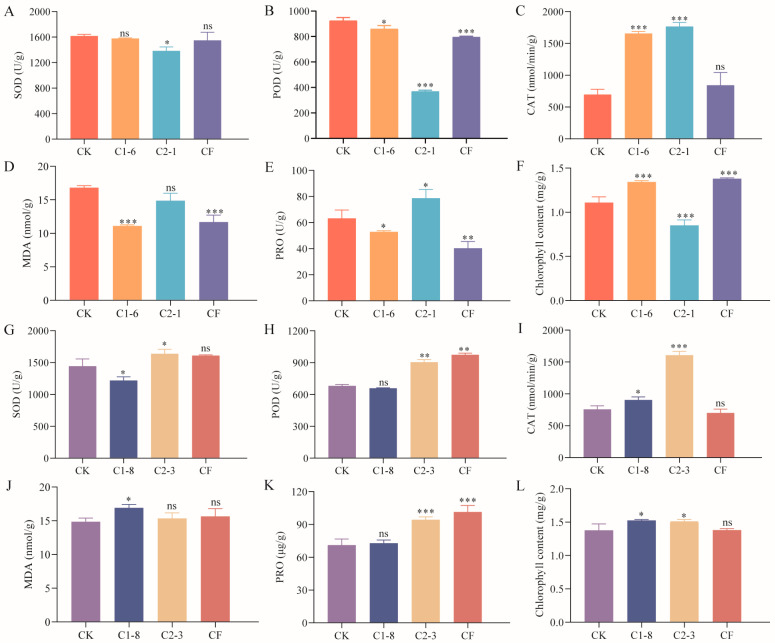
Effects of SMCs on enzyme activity and chlorophyll content in potted wheat. (**A**–**F**) Enzyme activity and chlorophyll content in spring wheat treated with C1-6 and C2-1. (**G**–**L**) Enzyme activity and chlorophyll content in winter wheat treated with C1-8 and C2-3. Data represent mean ± standard error (*n* = 10) and were analyzed using one-way ANOVA, ns, *p* > 0.05; *, *p* < 0.05; **, *p* < 0.01; ***, *p* < 0.001.

The results of enzyme activity and chlorophyll content analysis in wheat seedlings treated with SMCs revealed that, in spring wheat, C1-6 significantly enhanced catalase (CAT) enzyme activity and chlorophyll content, while markedly reducing malondialdehyde (MDA) and proline (PRO) enzyme activities (*p* < 0.001, *p* < 0.01, Figure 6C–F). Compared to CF, the effects of C1-6 on enzyme activity were more pronounced, suggesting that C1-6 exhibited superior efficacy. In winter wheat, C2-3 notably increased the activities of superoxide dismutase (SOD), peroxidase (POD), and CAT (*p* < 0.001, Figure 6F), as well as chlorophyll content (*p* < 0.05, Figure 6L). These enhancements were comparable to or even exceeded those achieved by CF.

## 3. Discussion

Salt stress poses a global challenge, severely impacting plant growth and serving as a major factor leading to reduced agricultural productivity. Salinity adversely affects germination, vigor, and yield, causing a drastic decline in plant productivity, particularly in arid and semi-arid regions [33]. In saline conditions, plant endophytes can be extracted and utilized to remediate soil degradation caused by salt stress [34,35,36]. *K. schrenkiana,* a halophyte belonging to the *Chenopodiaceae* family, has shown the potential to improve saline-alkaline soil properties, reduce soil salinity, and increase organic matter content. The endophytic microbes associated with this plant have developed unique ecological adaptations through long-term evolution [37].

In this study, endophytes isolated from *K. schrenkiana* in saline-alkaline soils of Xinjiang were characterized through 16S rRNA sequencing and phylogenetic tree analysis. The results revealed the complexity and diversity of the endophytic bacterial community of *K. schrenkianum*. The high abundance of *Actinobacteria* may be related to their specific niche in the plant endophytic environment, and bacteria from this phylum are known for their antibiotic production and plant growth-promoting properties [38,39]. The high abundance and diversity of *Proteobacteria* and *Firmicutes* further confirmed their ubiquity and importance in the plant microbiome. The low abundance of *Bacteroidetes* may indicate that their niche is small in this specific plant host or that they are less adaptable to environmental conditions than bacteria from other phyla [40].

Analysis at the genus level showed a high degree of differentiation of the microbial communities in the samples, which may be related to their specific functions inside the plant. For example, the genera *Bacillus* and *Streptomyces* have attracted much attention due to their potential roles in plant disease control and plant growth promotion [41,42]. The high abundance of these genera may be important for the growth and health of *K. schrenkianum*. In addition, the high abundance of *Alpha Proteobacterium* may indicate their important roles in plant–microbe interactions, including nutrient cycling and the activation of plant defense mechanisms. The high abundance of *Brevibacterium* and *Microbacterium* genera may be related to their functions in plant nutrient absorption and stress response.

These findings provide a basis for further exploring the functions of these endophytic bacteria in plant growth promotion and stress response, and provide a scientific basis for developing microbial-based plant health management strategies. Future studies can focus on the functional properties of these highly abundant genera and how they interact with host plants to promote plant health and productivity.

Further analyses of phosphate and potassium solubilization and ACC deaminase activity demonstrated that these strains play a critical role in promoting plant growth by enhancing nutrient availability and mitigating plant stress. These findings are consistent with previous studies, which reported that endophytes could enhance plant growth through multiple mechanisms [43]. Compared to PGPB from other sources, the unique evolutionary traits of *K. schrenkiana* endophytes in extreme environments endow them with superior functional capabilities, making them particularly well suited for applications under salt stress conditions.

Some strains demonstrated significant antagonistic effects, which may contribute to the suppression of soil pathogens, providing theoretical support for their potential use as biopesticides. However, the discovery of hemolytic properties in certain strains raises safety concerns. Further screening and genetic modifications will be necessary to enhance the safety and applicability of these strains in future development.

At present, synthetic functional microbiota has been widely used in agricultural production and biological control. For example, Hu used eight strains of *Pseudomonas* to form a synthetic microbiota to reduce the density of pathogens in the rhizosphere soil of tomatoes [44]. Niu used seven bacterial species in corn roots to inhibit the colonization of plant pathogenic fungi [45]. In terms of promoting plant growth, Durán [46] and Zhuang [47] screened strains that promote plant growth from the rhizosphere microbial communities of *Arabidopsis* roots and garlic, respectively; in terms of stress resistance, Castrillo [48] selected 35 strains in the roots of *Arabidopsis* and other cruciferous plants to construct a synthetic microbial community to enhance the activity of PHR1, the main regulator of phosphate stress response, and promote the integration of phosphate stress and immunity; Li [49] constructed a synthetic community composed of 13 bacterial strains to rescue *Astragalus* root rot by activating plant-induced systemic resistance. Schmitz [50] used five bacterial strains from the roots of the desert plant *Indigofera argentea* to construct a synthetic microbial community to protect tomatoes from high salt stress.

In this study, candidate strains were found to significantly improve the wheat germination rate, root length, and seedling growth through hydroponic experiments, thus confirming their potential to promote plant growth. A strategy that combined random selection with optimization effectively identified the most growth-promoting consortia for both spring and winter wheat. This method accounted for the synergistic effects among strains, which could arise from metabolic complementation and microenvironmental regulation. Random single-strain deletion experiments not only identified key strains but also validated their central roles in the cooperative interactions within the microbial community. The optimized consortia demonstrated superior growth-promoting effects in hydroponic experiments compared to unoptimized combinations. These findings highlight the importance of considering both the individual functionality of strains and the interactions and functional complementarities among strains when selecting strains for microbial consortia.

SMCs hold significant potential in promoting crop health by leveraging plant–microbe interactions to enhance plant growth and development and stress tolerance. Studies have shown that applying specific microbial agents, such as *Pantoea alhagi* LTYR-11Z, can increase soluble sugar content, reduce MDA accumulation, and slow chlorophyll degradation in wheat, thereby mitigating drought stress-induced damage and enhancing drought resistance [51]. Pot experiments further confirmed the stability and effectiveness of the selected microbial combinations under complex environmental conditions. The optimal SMCs (C1-6 and C2-3) not only increased wheat seedling length but also reduced oxidative stress by enhancing the activities of antioxidant enzymes (SOD, POD, CAT) and decreasing MDA levels. These findings suggest that such microbial consortia may promote plant growth by modulating antioxidant metabolic pathways. This conclusion is consistent with the existing literature, further providing additional support for the scientific mechanism through the regulation of metabolism and stress resistance [52,53,54,55]. Additionally, the observed increase in chlorophyll content indicates that the consortium may enhance photosynthetic efficiency, thereby supporting plant growth. Compared to commercial fertilizers, the microbial consortium demonstrated superior growth-promoting effects, presenting a promising alternative for the practical application of microbial resources. These results also underscore the dual benefits of reducing chemical fertilizer use while improving crop environmental adaptability and productivity, aligning with the goals of sustainable agriculture.

Despite the significant achievements in screening and optimizing salt-tolerant plant growth-promoting microbial consortia, this study has some limitations. First, the experiments were primarily conducted under hydroponic and pot cultivation conditions, leaving the stability and long-term efficacy of the SMCs in complex field environments unverified. Second, the specific mechanisms underlying the synergistic interactions within the microbial consortia remain unclear. Future research could employ multi-omics approaches, such as transcriptomics and metabolomics, to further elucidate how these consortia promote plant growth and enhance stress resistance. Additionally, exploring the potential applications of these microbial consortia in other crops could help broaden their utility.

Nonetheless, the SMCs developed in this study demonstrate significant potential for application in wheat cultivation in saline-alkaline soils, providing valuable insights into the agricultural utilization of microbial resources from extreme environments. These findings also offer critical data for the development of novel microbial formulations, which can help reduce the use of chemical fertilizers and promote more sustainable agricultural practices. In the future, further optimization of the microbial consortium and extensive field trials may expand its applicability, contributing to global food security and the ecological restoration of saline-alkaline lands.

## 4. Materials and Methods

### 4.1. Plant Samples Collection and Surface Sterilization

The samples of *K. schrenkianum* were collected from Hoxud County, Xinjiang Uygur Autonomous Region, China (91°45′42″ E, 40°39′75″ N). Surface sterilization was performed following the method described by Sun with slight modifications [56]. The plant tissues were first thoroughly rinsed with tap water, then sequentially immersed in 75% (*v*/*v*) ethanol for 1 min, 3.3% (*w*/*v*) sodium hypochlorite for 5 min, and again in 75% (*v*/*v*) ethanol for 30 s. Finally, the tissues were rinsed three times with sterile distilled water and dried using sterile filter paper for further processing.

### 4.2. Isolation and Cultivation of Strains

An amount of 1 g of surface-sterilized plant tissue was weighed and placed into 10 mL of sterile deionized water. The tissue was homogenized using a blender for 1 min. A 1 mL aliquot of the homogenized suspension was transferred to 9 mL of sterile deionized water to prepare a serial dilution. From each dilution gradient, 200 µL of the diluted suspension was spread onto 1/10 TSA medium and incubated at 28 °C for 3–15 days. After colony formation, initially screening was performed based on morphological characteristics, including shape, size, and color. Duplicate colonies with similar morphology were discarded, and distinct colonies were selected and transferred to slants of the respective isolation medium. These cultures were incubated in an inverted position at 28 °C for 5–10 days. Purified strains were preserved in glycerol tubes for further identification and for screening of stress resistance and functional properties.

### 4.3. DNA Extraction, Sequencing and Characterization

After sterilization, the plant tissue was ground, and genomic DNA was extracted using the cetyltrimethylammonium bromide (CTAB) method [57]. An amount of 1 g of surface-sterilized plant material was frozen in liquid nitrogen and then lyophilized before being ground into a fine powder using a mortar and pestle. The ground sample was quickly transferred to a tube containing 5 mL of 2 × CTAB extraction buffer [2% (*w*/*v*) CTAB, 100 mM Tris–HCl, 1.4 M NaCl, 20 mM EDTA, 1.5% (*w*/*v*) polyvinyl pyrrolidone (PVP), 0.5% (*w*/*v*) β-mercaptoethanol, pH 8.0], and incubated in a water bath at 60 °C for 30 min. Subsequently, 500 µL of chloroform and isoamyl alcohol (24:1) was added to each tube, followed by vigorous mixing to form an emulsion. The mixture was centrifuged at 11,900× *g* for 15 min at room temperature, and the upper phase containing DNA was transferred to a new 1.5 mL tube. This extraction was repeated twice. Next, 50 µL of 5 M KOAc and 400 µL of isopropyl alcohol were added to the supernatant, which was mixed gently by inversion. Genomic DNA was allowed to precipitate overnight at 4 °C and then centrifuged at 9200× g for 2 min. The obtained DNA was washed twice with 70% (*v*/*v*) ethanol and dried for 10 min in a SpeedVac (AES 1010; Savant, Holbrook, NY, USA). The DNA pellet was resuspended in 100 µL of TE buffer (10 mM Tris–HCl, 1 mM EDTA). DNA concentration and quality were measured using a NanoDrop 1000 spectrophotometer (Thermo Scientific, Wilmington, NC, USA). PCR amplification was performed using universal primers 27F (5′-AGAGTTTGATCCTGGCTCAG-3′) and 1492R (5′-GGTTACCTTGTTACGACTT-3′) for the 16S rRNA gene. The PCR products were verified by 1.5% agarose gel electrophoresis, purified, and sequenced by Sangon Biotech (Shanghai, China). The resulting sequences were submitted to the EzTaxon database (www.ezbiocloud.net, accessed on 1 August 2021) for similarity analysis to obtain the 16S rRNA gene sequence of the closest homologous reference strains. The 16S rRNA gene sequence for bacteria was uploaded to Figshare repository, resulting in a link for data (https://doi.org/10.6084/m9.figshare.28099616, accessed on 20 December 2024). ClustalW was used to align the 16S rRNA gene sequences, and the optimal nucleotide substitution model was determined by ModelTest-NG 0.1.6 software. Finally, MEGA 11 software was used to construct the phylogenetic tree using the neighbor-joining (NJ) method or the maximum likelihood (ML) method, and the node support of the tree was evaluated by 1000 bootstrap replicates.

### 4.4. Functional Characteristics of Endophytic Bacteria

#### 4.4.1. Salt Tolerance Assessment of Strains

Based on the 2003 provisional guidelines for ecological functional zoning issued by the National Environmental Protection Agency of China, the salinity threshold of severely saline-alkali soils in Northwest China is set at 2% [4]. Therefore, each strain was inoculated into 1/10 TSA medium containing 2% and 5% NaCl, respectively. The cultures were maintained at 30 °C and observed daily for 3–15 days to assess salt tolerance. Strains exhibiting robust tolerance were selected for further analysis.

#### 4.4.2. Phosphate-Solubilizing, Potassium-Releasing, and Nitrogen-Fixing Assays

The salt-tolerant strains identified above were inoculated onto solid phosphate-solubilizing medium and incubated at 30 °C for 72 h. The phosphate-solubilizing capability was preliminarily evaluated by calculating the ratio of the solubilization zone diameter to the colony diameter [58,59]. For assessing potassium-releasing ability, the strains were cultured on silicate bacteria medium. Colonies exhibiting a round, moist, smooth, and sticky surface, resembling water droplets with elasticity, were considered indicative of potassium-releasing activity. The nitrogen-fixing potential was preliminarily determined by observing the growth of strains on Ashby’s nitrogen-free medium, where positive nitrogen fixation was indicated by the presence of growth.

#### 4.4.3. Screening for ACC Deaminase-Positive Bacteria

The strains were inoculated into a nitrogen-free liquid medium and incubated at 30 °C with shaking at 200 rpm for 24 h. A 0.1 mL aliquot of medium was then transferred into 5 mL of DF liquid medium and incubated under the same conditions for 24 h. Subsequently, a 0.1 mL aliquot of medium was transferred to ADF liquid medium and incubated with shaking for an additional 48 h. This transfer step to ADF medium was repeated, and strains that could grow were identified as ACC deaminase-positive [60].

#### 4.4.4. Hemolysis Assay

The strains were spot-inoculated onto hemolysis agar and incubated at 30 °C for 3–7 days. Hemolytic activity was indicated by the formation of a 1–2 mm translucent hemolytic zone around the colonies [61].

#### 4.4.5. Antagonism Test of Strains

The selected strains were subjected to pairwise antagonism tests. After liquid culturing, the strains were cross-inoculated onto agar plates by streaking. The plates were then incubated at 30 °C for 24 h. Antagonistic activity between the strains was assessed by the presence of clear inhibition zones at the intersection of the streaks [62].

### 4.5. Study on the Plant Growth-Promoting Functions of Endophytic Bacteria

Strains with good salt-alkali tolerance and no pathogenicity were selected for this study. The selected strains were inoculated into 1/10 TSA medium containing 2% NaCl and cultured until reaching the exponential growth phase. Cells were harvested by centrifugation, washed once with PBS, and resuspended in 10 mM MgCl_2_ solution. The OD_600_ of each cell suspension was adjusted to 0.1. When forming SMC, various bacteria were mixed in equal proportions according to the composition, and the mixture was diluted to an OD_600_ of 0.02.

Wheat seeds from two commonly cultivated varieties in Xinjiang, “Xin Chun 26” and “Xin Dong 18”, were surface-sterilized by treating with 3% NaClO solution for 1 min, repeated three times, followed by three washes with sterile water. The seeds were then placed in sterile Petri dishes (100 mm × 100 mm) containing sterile filter paper. The treatments included a control group with 1.2% NaCl and various SMC groups with 1.2% NaCl. Each treatment consisted of three technical replicates and three biological replicates, with 20 seeds per Petri dish for germination.

After approximately 10 days, when the third true leaf emerged from the seedlings, growth indicators, including germination rate, root length, and seed length, were measured to preliminarily evaluate the growth-promoting effects.

### 4.6. Construction of SMCs and Validation in Wheat

Based on the abundance of dominant taxa within the endophytic microbial community, the co-occurrence network relationships among community members, and functional prediction results [63], 11 representative strains from dominant genera were selected. The freshly cultured strains were diluted to an OD_600_ of 0.02 and then mixed in equal proportions to construct SMCs. Various treatments were designed with different strain combinations, including treatments containing all selected strains, treatments with one strain removed, and combinations with a gradient reduction in the number of strains. While varying the community richness, the total biomass was kept constant. Germination experiments were then conducted on wheat using these different synthetic microbial community combinations to validate their effects on salt tolerance according to previously reported methods.

### 4.7. Wheat Pot Experiment

The soil used in this potted plant experiment was taken from the saline-alkali land in Qitai County, Xinjiang, with a salinity level ranging from 1% to 1.5%. The pre-established synthetic microbial communities were used to soak the seeds of both spring and winter wheat. During the wheat growth, a synthetic microbial agent with an OD_600_ of 0.2 was applied as inoculum. Negative and positive controls were set up using water and commercial fertilizers (Yeshengwang Biotechnology, Beihai, China), respectively. After 20 days of wheat growth, morphological changes and seedling length were observed. In addition, 10 wheat seedlings were randomly selected, and the activities of SOD, POD, CAT, MDA, PRO, and chlorophyll were measured by Suzhou Comin Biotechnology Company (Suzhou, China) [64,65,66].

### 4.8. Statistical Analyses

Data are expressed as mean ± standard error (SE) and analyzed using SPSS 25.0 (IBM Corp. Armonk, NY, USA) and Graphpad Prism 8.0 (San Diego, CA, USA). One-way ANOVA and Tukey’s test were employed to determine the significant differences among multiple groups. In all tests, a *p* < 0.05 was considered statistically significant.

## 5. Conclusions

This study highlights the promising potential of endophytic bacterial strains isolated from *K. schrenkianum*, a halophyte adapted to saline-alkaline environments, in promoting plant growth under salt stress. SMCs composed of these strains significantly enhanced wheat growth, including improvements in seed germination, root development, and seedling vigor. Furthermore, the SMCs demonstrated the ability to mitigate oxidative stress and improve antioxidant enzyme activities, indicating their potential to support wheat growth in saline soils. These results suggest that microbial consortia derived from extreme environments could serve as an eco-friendly and sustainable alternative to chemical fertilizers, contributing to enhanced agricultural productivity and resilience in saline-affected regions. Future research may further explore the underlying mechanisms and scalability of such microbial applications in diverse agricultural contexts.

## Figures and Tables

**Table 1:** Screening strain information.

Number of Strains	The Taxonomic Generic (Species) Name of the Strains
2	*Gordonia hydrophobica*
3	*Streptomyces monticola*
6	*Planomicrobium okeanokoites*
8	*Microbacterium amylolyticum*
9	*Pseudomonas stutzeri*
10	*Brachybacterium alimentarium*
13	*Luteimonas huabeiensis*
14	*Bacillus australimaris*
16	*Microbacterium thalassium*
18	*Brevibacterium epidermidis*
37	*Bacillus cabriales* *ii*

## Data Availability

The original contributions presented in this study are included in the article/Supplementary Materials; further inquiries can be directed to the corresponding author/s.

## Supplementary Materials

The supporting information can be downloaded at www.mdpi.com/article/10.3390/ijms26020860/s1.
