# Supplementary material for: Application of Synthetic Microbial Communities of Kalidium schrenkianum in Enhancing Wheat Salt Stress Tolerance
Source: Int J Mol Sci. 2025 Jan 20;26(2):860. doi: 10.3390/ijms26020860 (PMC11765726; doi:10.3390/ijms26020860)
Supplement: Supplementary file 1 [file ijms-26-00860-s001.zip › ijms-3375918-supplementary.pdf]

**Table S1.** Culturable bacteria endophytic to *K. schrenkianum*

| Classification Genus (species) name | Isolate strain number |
|---|---|
| *Agrococcus citreus* | 76 |
| *Agromyces ramosus* | 2-19 |
| *Alcaligenes faecalis* | 1, CK2 5-R |
| *Alpha proteobacterium* | 137, 139, 140, 141, 143, 146, 147, 148 |
| *Arthrobacter bussei* | 3 |
| *Arthrobacter crystallopoietes* | 34, 2-12 |
| *Arthrobacter pascens* | 2-10, 2-11, 2-16 |
| *Bacillus cabrialesii* | 21 |
| *Bacillus megaterium* | 22 |
| *Bacillus rugosus* | 2-8, 25, 41, 98, 150, 171, 72 |
| *Bacillus thuringiensis* | 129, 151 |
| *Bacillus vallismortis* | 172 |
| *Bacillus australimaris* | 24 |
| *Brachybacterium alimentarium* | 17, 114 |
| *Brachybacterium massiliense* | 158 |
| *Bradyrhizobium lupini* | 155 |
| *Brevibacterium avium* | 50 |
| *Brevibacterium epidermidis* | 42, 59, 77, 99, 103, 105, 126, 43, 80, 82, 101, 118 |
| *Brevibacterium linens* | 61 |
| *Brevibacterium metallicus* | 36, 124 |
| *Brevibacterium pigmentatum* | 130 |
| *Brevibacterium sediminis* | 11, 13, 16, 44, 46, 52, 58, 65, 84, 85, 90, 95, 109, 112, 18, 37, 45, 57, 107 |
| *Brevibacterium siliguriense* | 47, 54, 66, 83, 102, 127, 91, 116 |
| *Brevundimonas diminuta* | 35, 48, 78, 87, 89, 100, 104, 122, 123 |
| *Brevundimonas naejangsanensis* | 51, 53, 64, 88, 94, 106, 119 |
| *Brucella pseudogrignonensis* | 120, 128, 108 |
| *Chelativorans multitrophicus* | 4 |
| *Chitinophaga japonensis* | CK25-1R-H2 |
| *Devosia nitrariae* | 176 |
| *Enterovirga rhinocerotis* | 170 |
| *Fictibacillus nanhaiensis* | 2-2, 2-1, 2-4, 2-5 |
| *Fictibacillus phosphorivorans* | 7, 8, 9, 70, 134, 169, 2-6 |
| *Georgenia muralis* | 2-14 |
| *Gordonia hydrophobica* | 39 |
| *Kocuria himachalensis* | 2-21, 2-22 |
| *Leucobacter denitrificans* | 56, 92 |
| *Leucobacter luti* | 15 |
| *Luteimonas huabeiensis* | 159 |
| *Mammaliicoccus sciuri* | 2-9 |
| *Microbacterium amylolyticum* | 49, 62, 86, 115, 38, 79 |

| | |
|---|---|
| *Microbacterium sp.* | 121 |
| *Microbacterium thalassium* | 55 |
| *Moraxella osloensis* | 75 |
| *Novosphingobium colocasiae* | 136, 138, 144, 152 |
| *Novosphingobium soli* | 133, 177 |
| *Ochrobactrum pseudogrignonense* | 2, 12, 14 |
| *Paenibacillus ourofinensis* | 113 |
| *Parapedobacter indicus* | 166, 173 |
| *Parapedobacter luteus* | 168, CK2, 1-5R |
| *Pseudarthrobacter oxydans* | 19 |
| *Pseudarthrobacter polychromogenes* | 60, 2-18, 2-7 |
| *Pseudarthrobacter siccitolerans* | 154 |
| *Pseudomonas stutzeri* | 6, 10, 69, 5 |
| *Pseudonocardia cypriaca* | 175 |
| *Pseudoxanthomonas wuyuanensis* | 68, 132, 2-3 |
| *Ramlibacter alkalitolerans* | 2-20 |
| *Roseomonas vinacea* | 174 |
| *Sinorhizobium meliloti* | 30, 31, 32, 26, 27, 33, 73 |
| *Sphingomonas rhizophila* | 178 |
| *Staphylococcus warneri* | 160, 2-15 |
| *Streptomyces monticola* | 74 |
| *Streptomyces seymenliensis* | 2-17, 2-13, 71, 20 |
| *Unidentified bacterium* | 145, CK21-1R |
